# Construction of a risk prediction model for postoperative pneumonia based on the prognostic nutritional index and analysis of related factors in patients with intracerebral hemorrhage

**DOI:** 10.3389/fnut.2025.1639230

**Published:** 2025-09-12

**Authors:** Tingxuan Wang, Haitao Wu, Yue Bao, Bin Lu, Luo Li

**Affiliations:** ^1^Qingdao Medical College, Qingdao University, Qingdao, Shandong, China; ^2^Department of Neurosurgery, Qingdao Hospital, University of Health and Rehabilitation Sciences (Qingdao Municipal Hospital), Qingdao, Shandong, China

**Keywords:** cerebral hemorrhage, postoperative pneumonia, nutritional index, prognostic nutritional index, prediction model

## Abstract

**Introduction:**

Postoperative pneumonia (POP) is a common complication following hematoma extraction in patients with cerebral hemorrhage, contributing to poor prognosis. Prognostic nutritional index (PNI), a composite index combining serum albumin (a marker of nutritional status) and lymphocyte count (a marker of immune function), reflects both nutritional reserve and immune competence. Impaired nutritional status and immune dysfunction are key drivers of postoperative infections, making PNI a theoretically plausible indicator for predicting POP. This study aimed to explore the relationship between POP and nutritional indices (with a focus on PNI) after hematoma clearance and to develop a predictive model for POP.

**Methods:**

A retrospective analysis was conducted on 325 patients who underwent hematoma removal, including 133 patients diagnosed with POP. The PNI was calculated using the formula: PNI = 5 × lymphocyte count (×10^9^/L) + serum albumin (g/L). Univariate and multivariate logistic regression analyses were performed to identify independent risk factors for POP. The performance of the predictive model was evaluated using the area under the receiver operating characteristic curve (AUC), internal validation, and visualization via a Nomogram.

**Results:**

Significant POP risk factors: low PNI (*p* < 0.001, OR = 0.84, 95%CI 0.77–0.90), hypoproteinemia (*p* = 0.008, OR = 2.91), low admission GCS (*p* = 0.009, OR = 2.92), tracheotomy (*p* = 0.002, OR = 3.92), and obstructive lung diseases (*p* = 0.014, OR = 4.22). The model (incorporating these factors) had an AUC of 0.87, passed validation, and was visualized as a Nomogram. This is the first identification of PNI as a POP risk factor in this population.

**Conclusion:**

The predictive model, which integrates PNI and four other clinical factors, demonstrates favorable discriminative ability in identifying patients at high risk of POP following hematoma extraction for cerebral hemorrhage. By quantifying the risk of POP preoperatively, this model can assist clinicians in stratifying patients, prioritizing targeted preventive interventions (such as nutritional optimization or respiratory care) for high-risk individuals, and thereby contributing to the reduction of postoperative complications.

## Introduction

Intracerebral hemorrhage (ICH) is a neurological emergency characterized by high morbidity and mortality, with a global incidence ranging from 6.5 to 19.6% and a 30-day mortality rate as high as 33% (1, 2). Surgical evacuation of intracerebral hematomas significantly improves patients’ survival outcomes by reducing intracranial pressure, relieving the mass effect of the hematoma, and mitigating the neurotoxicity of blood degradation products (1). Among the multiple factors influencing outcomes in patients with ICH, postoperative pneumonia (POP)—a common yet severe complication—poses a substantial threat to prognosis. This complication not only delays patient recovery and exacerbates disease progression but also adversely affects both short- and long-term outcomes, while being closely associated with readmission rates and mortality (3). Thus, early identification of high-risk individuals for POP following ICH and implementation of targeted preventive strategies hold significant practical value for improving patients’ clinical outcomes.

To date, research on risk prediction of POP has predominantly focused on demographic features, history of comorbidities, surgical approaches, and postoperative interventions (4–6), with relatively limited exploration into indicators related to patients’ nutritional status. Accumulating evidence has confirmed that optimization of nutritional indices through dietary interventions can effectively reduce the risk of stroke (7). In this context, the prognostic nutritional index (PNI)—a quantitative metric that comprehensively assesses both nutritional reserve and immune function—holds unique application potential in the present study (8). By integrating serum albumin levels and peripheral blood lymphocyte counts into its calculation, PNI enables simultaneous evaluation of the body’s protein reserve capacity and immune defense function. Therefore, this study aims to incorporate multiple nutritional indices, including PNI, to systematically investigate their associations with POP following intracerebral hemorrhage. Furthermore, a risk prediction model for POP will be constructed based on these nutritional indices, with the goal of providing clinicians with a reference for preoperative risk stratification and targeted prevention, ultimately improving patient prognosis and quality of life.

## Data from Patients

Patients with intracerebral hemorrhage admitted to Qingdao Municipal Hospital from January 2014 to December 2024 were retrospectively enrolled, and their electronic medical record data were extracted. The Institutional Review Board was approved by the Ethics Committee of Qingdao General Hospital. All procedures were carried out in accordance with the guidelines of the Helsinki Declaration. Inclusion criteria: (1) Confirmed as intracerebral hemorrhage by cranial computed tomography (CT) after admission (2); Underwent hematoma evacuation (3); With complete and traceable clinical data. Exclusion criteria: (1) Pre-admission confirmed diagnosis of pneumonia (including community-acquired or hospital-acquired pneumonia) (2); Confirmed advanced malignant tumors, severe liver impairment (e.g., Child-Pugh class C) or renal impairment (e.g., CKD stage 5).

### Demographic characteristics

gender, age, Hypertension (HTN) was defined as a documented prior diagnosis of hypertension (with medical records) or an admission systolic blood pressure ≥140 mmHg and/or diastolic blood pressure ≥90 mmHg (based on the first blood pressure measurement upon admission). Data were extracted from past medical history records and admission vital sign monitoring. Diabetes mellitus (DM) was defined as a documented prior diagnosis of diabetes (with medical records) or an admission fasting blood glucose ≥7.0 mmol/L (based on preoperative fasting glucose tests). Data were retrieved from past medical history and laboratory test records. Coronary heart disease (CHD) was confirmed based on a documented prior diagnosis (e.g., myocardial infarction, angina pectoris, etc., with medical records), with data extracted from the past medical history section of electronic medical records. Obstructive lung disease (OLD) included chronic bronchitis, pulmonary emphysema, bronchiectasis, and chronic obstructive pulmonary disease (COPD), defined as a documented prior diagnosis supported by imaging or pulmonary function tests. Data were obtained from past medical history and respiratory specialist records. Smoking history was defined as a self-reported or medically documented long-term smoking history (cumulative smoking ≥100 cigarettes for more than 6 months), with data extracted from admission history-taking records. Glasgow Coma Scale (GCS) score at admission: within 1 h after admission, the patients were scored according to the standard score of eye-opening reaction, language reaction and motor reaction by the medical staff, and divided into three levels: Grade I (13 to 15 points, mild disturbance of consciousness), grade II (9 to 12 points, moderate disturbance of consciousness), and grade III (3 to 8 points, severe disturbance of consciousness) were recorded from neurological assessment at admission; surgical type (craniotomy hematoma removal, minimally invasive hematoma removal), operation duration, intraoperative blood loss (IbL), ventilator assisted time (VAT), tracheotomy, postoperative pleural effusion (PPE), postoperative coma time (PCT).

### Nutritional index

Through literature review, we collected nutritional index of patients (All data were taken from preoperative data of patients), including: total protein (TP), hemoglobin (HGB), prognostic nutritional index (PNI): 5 × lymphocyte count (10^9^/L) + serum albumin (g/L), blood glucose before breakfast (BGBB), platelet count (PLT), lymphocyte count (LC), anemia (male < 120 g/L; female < 110 g/L), hypoproteinemia (albumin < 35 g/L or total protein < 60 g/L).

The US Centers for Disease Control and Prevention defines POP as follows: a new-onset pneumonia in surgical patients within 30 days after surgery, including pneumonia that occurs after discharge but within 30 days postoperatively. The diagnosis meets the following three criteria: 1. Chest X-ray indicates new or progressive persistent infiltrates, consolidations, or cavitations in the lung. 2. Peripheral blood white blood cell (WBC) count >12 × 10^9^/L or <4 × 10^9^/L; or, in patients over 70 years of age, altered mental status without other identified causes. 3. At least two of the following:

- New onset of purulent sputum or changes in the nature and quantity of sputum;
- New onset of cough, tachypnea, dyspnea, or exacerbation of existing symptoms;
- Deterioration in gas exchange (increased oxygen demand, requirement for mechanical ventilation) (9).

### Statistical analysis

Statistical analyses were performed using SPSS Statistics 27.0 and R 4.2 software. Patients were divided into two groups based on the presence or absence of POP. Categorical variables were presented as frequencies, and differences between groups were compared using the chi-square test or Fisher’s exact test. For continuous variables, according to the results of the Kolmogorov–Smirnov test, those with a normal distribution were expressed as mean ± standard deviation (mean ± SD) and compared using the t-test; those with a non-normal distribution were presented as median and interquartile range (IQR), and between-group differences were analyzed using the Mann–Whitney U test. All potential risk factors were included in univariate and multivariate binary logistic regression analyses. Restricted cubic splines were used to flexibly model and visualize the relationships between risk factors and POP. Predictive models were constructed with the receiver operating characteristic (ROC) curve and area under the curve (AUC) as indicators of discriminative ability. A 1000-repeat bootstrap resampling strategy was implemented for optimism correction, and calibration was confirmed using the Hosmer-Lemeshow test. The 325 patients were randomly divided into a training set and a validation set at a ratio of 7:3 for internal validation of the predictive model. To better apply the new risk factors in clinical practice, a Nomogram prediction model was established. Decision curve analysis (DCA) was used for comprehensive evaluation of POP. All statistical tests were two-tailed, and a *p*-value < 0.05 was considered statistically significant.

## Results

Per the inclusion and exclusion criteria, a total of 325 patients were enrolled in this study. 133 patients were diagnosed with POP, and 192 patients were diagnosed with non-POP. Table 1 summarizes the preoperative demographic characteristics and nutritional indices of patients with intracerebral hemorrhage. The mean age of the patients was 59.07 ± 13.97 years, with a male predominance (68.62%). The PNI was 42.22 ± 6.09; HGB was 128.00 (113.00, 141.00) g/L; BGBB was 6.66 (5.41, 8.98) mmol/L; and PLT was 208.00 (161.00, 255.00) × 10^9^/L. Hypoproteinemia was observed in 180 cases (55.38%), and anemia in 94 cases (28.92%). Of the 325 patients, 133 (40.92%) developed postoperative pneumonia (POP). In POP patients, PNI (38.24 ± 5.27 vs. 44.97 ± 5.01), HGB (122.00 [107.00, 138.00] g/L vs. 133.00 [119.00, 143.00] g/L), TP (60.03 [54.81, 64.39] g/L vs. 65.87 [61.86, 70.10] g/L), and PLT (188.00 [144.00, 253.00] × 10^9^/L vs. 218.00 [173.75, 256.25] × 10^9^/L) were all lower than those in non-POP patients (Table 1).

**Table 1:** Baseline variables as well as univariate binomial logistic regression analysis in patients with POP.

Variables	Total (*n* = 325)	POP		*p*-value (Univariate)
		Absent (*n* = 192)	Present (*n* = 133)	
Age, (year)	59.07 ± 13.97	57.13 ± 14.15	61.88 ± 13.27	**0.003**
Sex, n (%)				0.061
Male	223 (68.62)	124 (64.58)	99 (74.44)	
Female	102 (31.38)	68 (35.42)	34 (25.56)	
GCS at admission, n (%)				**<0.001**
I (13–15)	110 (33.85)	77 (40.10)	33 (24.81)	
II (9–12)	131 (40.31)	84 (43.75)	47 (35.34)	
III (3–8)	84 (25.85)	31 (16.15)	53 (39.85)	
HTN, n (%)				0.896
No	133 (40.92)	78 (40.62)	55 (41.35)	
Yes	192 (59.08)	114 (59.38)	78 (58.65)	
DM, n (%)				0.121
No	276 (84.92)	168 (87.50)	108 (81.20)	
Yes	49 (15.08)	24 (12.50)	25 (18.80)	
CHD, n (%)				0.598
No	308 (94.77)	183 (95.31)	125 (93.98)	
Yes	17 (5.23)	9 (4.69)	8 (6.02)	
OLD, n (%)				**<0.001**
No	290 (89.23)	184 (95.83)	106 (79.70)	
Yes	35 (10.77)	8 (4.17)	27 (20.30)	
Smoke, n (%)				0.030
No	263 (80.92)	163 (84.90)	100 (75.19)	
Yes	62 (19.08)	29 (15.10)	33 (24.81)	
Anemia, n (%)				**<0.001**
No	231 (71.08)	151 (78.65)	80 (60.15)	
Yes	94 (28.92)	41 (21.35)	53 (39.85)	
TP (g/L)	63.67 (58.78, 68.07)	65.87 (61.86, 70.10)	60.03 (54.81, 64.39)	**<0.001**
HGB (g/L)	128.00 (113.00, 141.00)	133.00 (119.00, 143.00)	122.00 (107.00, 138.00)	**<0.001**
BGBB (mmol / L)	6.66 (5.41, 8.98)	6.35 (5.35, 8.79)	7.00 (5.55, 9.05)	0.143
PLT (10^9^/L)	208.00 (161.00, 255.00)	218.00 (173.75, 256.25)	188.00 (144.00, 253.00)	0.222
PNI	42.22 ± 6.09	44.97 ± 5.01	38.24 ± 5.27	**<0.001**
Craniotomy, n (%)				0.611
No	83 (25.54)	51 (26.56)	32 (24.06)	
Yes	242 (74.46)	141 (73.44)	101 (75.94)	
Surgical time (min)	150.00 (96.00, 210.00)	150.00 (90.00, 200.00)	169.00 (105.00, 220.00)	0.579
IBL (ml)	100.00 (20.00, 300.00)	97.50 (20.00, 300.00)	100.00 (20.00, 300.00)	0.154
Tracheotomy, n (%)				**<0.001**
No	265 (81.54)	177 (92.19)	88 (66.17)	
Yes	60 (18.46)	15 (7.81)	45 (33.83)	
VAT>96H, n (%)				**<0.001**
No	265 (81.79)	172 (89.58)	93 (70.45)	
Yes	59 (18.21)	20 (10.42)	39 (29.55)	
PPE, n (%)				**<0.001**
No	169 (52.00)	116 (60.42)	53 (39.85)	
Yes	156 (48.00)	76 (39.58)	80 (60.15)	
PCT>3 day, n (%)				**<0.001**
No	168 (51.69)	117 (60.94)	51 (38.35)	
Yes	157 (48.31)	75 (39.06)	82 (61.65)	

The bold values indicate that there are statistically significant differences in the *p* values. HTN, hypertension; DM, diabetes mellitus; CHD, coronary heart disease; OLD, obstructive lung diseases; BGBB, Blood glucose before breakfast; IbL, intraoperative blood loss; VAT, ventilator assisted time; PPE, postoperative pleural effusion; PCT, postoperative coma time; TP, Total protein; HGB, Hemoglobin.

Univariate and multivariate binary logistic regression analyses were performed to examine the associations between POP and clinical variables. Per univariate analysis (Table 1), the following variables showed statistically significant differences with POP: age (*p* = 0.003), GCS at admission (*p* < 0.001), OLD (*p* < 0.001), anemia (*p* < 0.001), hypoproteinemia (*p* < 0.001), PNI (*p* < 0.001), TP (*p* < 0.001), HGB (*p* < 0.001), VAT > 96 h (*p* < 0.001), tracheotomy (*p* < 0.001), PPE (*p* < 0.001), and PCT > 3 days (*p* < 0.001). Subsequently, variables with statistical significance in univariate analysis (*p* < 0.05) were included in multivariate regression analysis, and a backward stepwise selection method was applied to identify statistically significant factors in the multivariate model. As shown in Table 2, the results revealed that PNI (*p* < 0.001, OR = 0.84, 95% CI: 0.77–0.90), GCS (*p* = 0.009, OR = 2.92, 95% CI: 1.31–6.51), OLD (*p* = 0.014, OR = 4.22, 95% CI: 1.34–13.32), hypoproteinemia (*p* = 0.008, OR = 2.91, 95% CI: 1.32–6.41), and tracheotomy (*p* = 0.002, OR = 3.92, 95% CI: 1.67–9.18) remained independent risk factors for POP in patients with intracerebral hemorrhage. For these five factors, tolerance was > 0.5 and variance inflation factor (VIF) < 10, indicating no multicollinearity among them (Table 2).

**Table 2:** Multivariate binomial logistic regression analysis as well as analysis of collinearity in patients with POP.

Variables	*p*-value (Multivariate)	OR (95%CI)	Tolerance	VIF
PNI	**<0.001**	0.84 (0.77–0.90)	0.614	1.629
GCS	**0.009**	2.92 (1.31–6.51)	0.945	1.058
I (13–15)				
II (9–12)				
III (3–8)				
OLD	**0.014**	4.22 (1.34–13.32)	0.973	1.028
No				
Yes				
Hypoproteinemia	**0.008**	2.91 (1.32–6.41)	0.660	1.515
No				
Yes				
Tracheotomy	**0.002**	3.92 (1.67–9.18)	0.927	1.079
No				
Yes				
VAT>96H	0.629	1.24 (0.52–2.98)		
No				
Yes				
PPE	0.667	1.15 (0.61–2.16)		
No				
Yes				
PCT>3 day	0.637	1.16 (0.62–2.20)		
No				
Yes				
Smoke	0.280	1.63 (0.67–3.93)		
No				
Yes				
Anemia	0.368	1.56 (0.60–4.06)		
No				
Yes				
Age	0.867	1.00 (0.98–1.02)		
TP	0.617	0.99 (0.93–1.04)		
HGB	0.484	1.01 (0.99–1.03)		

The bold values indicate that there are statistically significant differences in the *p* values. OLD, obstructive lung diseases; VAT, ventilator assisted time; PPE, postoperative pleural effusion; PCT, postoperative coma time; TP, Total protein; HGB, Hemoglobin.

## Restricted cubic spline analysis of the relationship between continuous variables and POP

**Figure 1:**
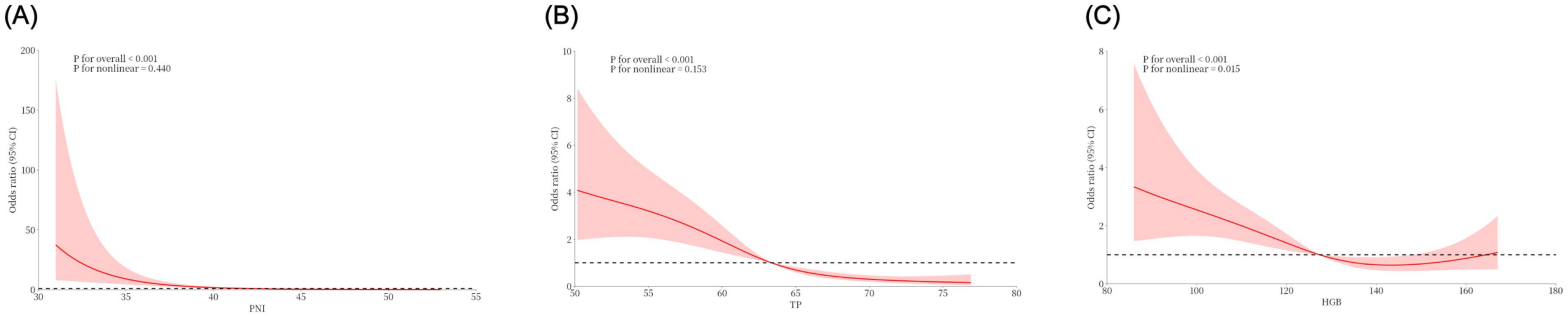
The association of three continuous variables, PNI **(A)**, TP **(B)**, and HGB **(C)**, with the risk of postoperative pneumonia (POP) was visualized by restricted cubic splines.

Using RCS, we visualized the relationship between continuous variables with statistical associations and POP. The results are as follows: 1 PNI (Figure 1A): The overall *p*-value < 0.001 indicates a significant statistical association between PNI and the risk of POP. A nonlinear *p*-value of 0.440 suggests insufficient evidence to support a nonlinear relationship between PNI and POP risk. The curve shows that the odds ratio (OR) first decreases rapidly and then stabilizes as PNI increases, indicating that lower PNI levels are associated with a higher risk of POP, making low PNI a potential risk factor for POP. 2 TP (Figure 1B): The overall *p*-value < 0.001 indicates a significant association between TP and POP risk. A nonlinear p-value of 0.153 suggests no clear evidence of a nonlinear relationship. The curve shows a downward trend in OR as TP increases, implying that low total protein may elevate the risk of POP, though the strength of this association requires further evaluation in clinical practice. 3. HGB (Figure 1C): The overall *p*-value < 0.001 confirms a significant association between HGB and POP risk. A nonlinear p-value of 0.015 indicates statistical evidence supporting a nonlinear relationship (i.e., the association is not simply linear). The curve shows that OR tends to increase when HGB is extremely low or high (with relative stability in the intermediate range), suggesting that clinical attention should be paid to abnormal HGB levels (either too low or too high) as potential contributors to POP risk.

## Performance evaluation and validation of predictive models for postoperative pneumonia in patients with intracerebral hemorrhage

**Figure 2:**
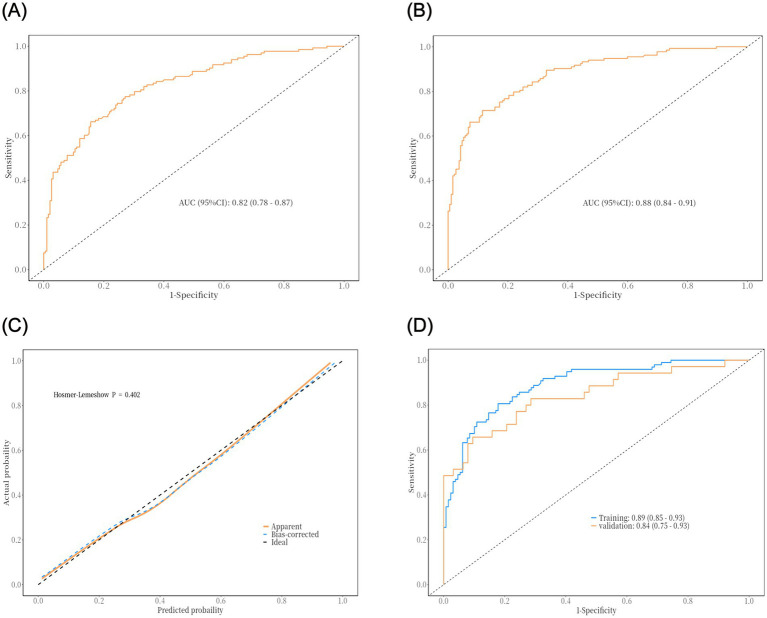
The results of ROC curve analysis and internal validation of the prediction model: **(A)** ROC curve of univariate model (predicted by PNI alone); **(B)** ROC curve of combined model (OLD, GCS score, hypoproteinemia, PNI and tracheotomy); **(C)** Model calibration curve (Hosmer-Lemeshow test); **(D)** ROC curve of internal validation (training set vs. validation set).

**Figure 3:**
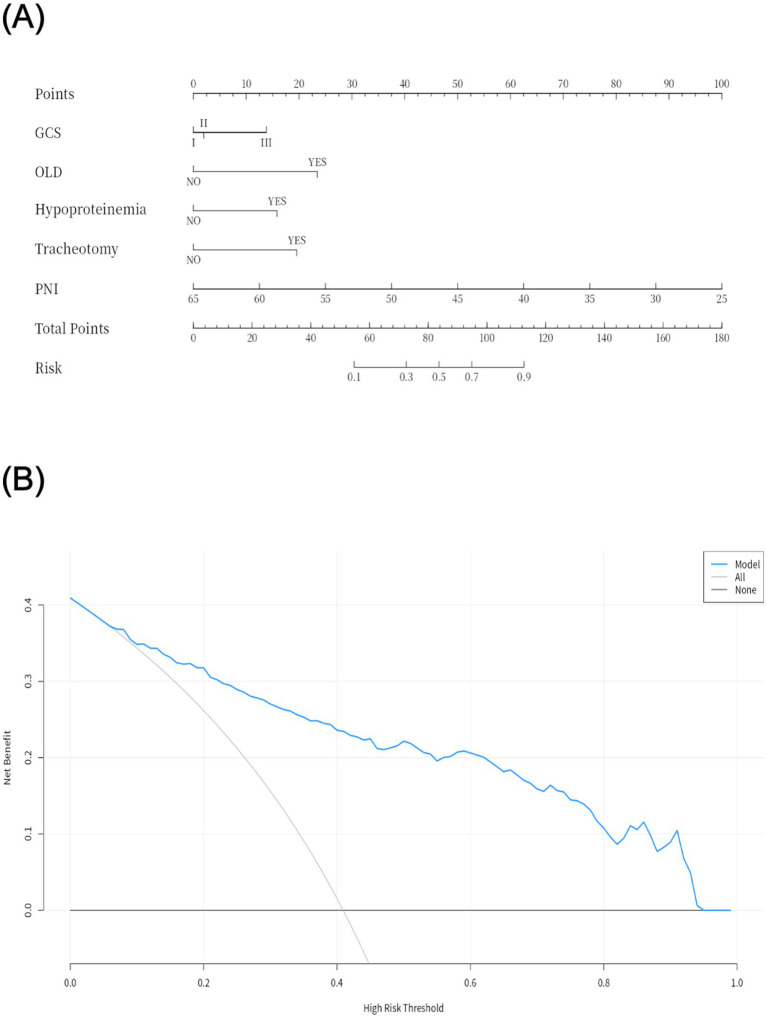
Visualization of the prediction model **(A)** and decision curve analysis **(B)**. GCS: I (13–15), II (9–12), III (3–8); OLD, obstructive lung diseases; PNI, Prognostic nutritional index.

The PNI is a novel composite metric that integrates immune and nutritional factors. Statistical results demonstrated that PNI remained statistically significant in both univariate and multivariate binary logistic regression analyses. When PNI was incorporated into ROC curve analysis (Figure 2A), the AUC was 0.82 (95% CI: 0.78–0.87), with a sensitivity of 84%, specificity of 66%, and an optimal cutoff value of 39.81—indicating favorable performance of the prediction model. To improve the model’s predictive efficacy, OLD, GCS score, hypoproteinemia, and tracheotomy were further included in ROC curve analysis (Figure 2B). The combined model yielded an AUC of 0.87 (95% CI: 0.84–0.91), with a sensitivity of 64.7% and specificity of 95.3%, confirming its robust predictive ability. A Delong test comparing the two models showed *p* > 0.05, indicating no significant disparity in their predictive capacity. Nevertheless, the combined model retains higher clinical utility due to its markedly improved specificity, more comprehensive risk assessment, and stronger potential for intervention—serving as a more practical tool for the precise prevention of POP in patients with intracerebral hemorrhage. Subsequently, a 1,000-repetition bootstrap resampling strategy was applied for optimism correction. The Hosmer-Lemeshow test yielded a *p*-value of 0.402 (Figure 2C), and the calibration curve closely overlapped with the ideal reference line—further validating the model’s excellent predictive performance. For internal validation, the 325 patients were randomly split into a training set and a validation set at a 7:3 ratio, with ROC curve analysis used for verification. Results showed an AUC of 0.89 (95% CI: 0.85–0.93) in the training set and 0.84 (95% CI: 0.75–0.93) in the validation set (Figure 2D). The prediction model was visualized as a nomogram (Figure 3A). DCA (Figure 3B) revealed that when an individual’s threshold probability falls between 7 and 97%, the screening strategy based on the POP risk nomogram yields a greater net benefit than either “no screening” or “screening all” strategies.

## Discussion

Compared with patients in other surgical specialties, neurosurgical patients exhibit a significantly higher prevalence of POP. This is attributed to multiple factors, including prolonged coma, extended bed rest, and impaired cough and swallowing reflexes secondary to cerebrovascular diseases (10, 11). In neurosurgery, postoperative infections rank among the most life-threatening complications, with POP being a primary subtype (12, 13). Studies have demonstrated that patients with POP experience longer ICU stays, extended postoperative hospitalizations, higher readmission rates, increased healthcare costs, and elevated mortality compared to those without POP (14–17). Thus, implementing preventive measures during the perioperative period to mitigate POP is both reasonable and imperative. This study focuses on investigating the impact of nutritional indicators and other pneumonia-related risk factors on the development of POP in patients with intracerebral hemorrhage. The results indicate that both univariate and multivariate binary logistic regression analyses identified PNI, OLD, GCS score, hypoproteinemia, and tracheotomy as independent risk factors for POP in this patient population. The prediction model constructed based on these five factors shows favorable predictive efficacy for POP following intracerebral hemorrhage surgery. Notably, this study is the first to propose that PNI can serve as a risk indicator for POP in patients with intracerebral hemorrhage, thereby providing a novel dimension for risk assessment in this field.

The PNI, a novel nutritional metric proposed by Japanese scholar Onodera, integrates albumin (a nutritional marker) and lymphocyte count (LC,an immune marker) as a comprehensive indicator of nutritional status. To date, PNI has been validated as a prognostic predictor for cardiovascular mortality, all-cause mortality, and outcomes in gastrointestinal malignancies, nasopharyngeal carcinoma, and esophageal cancer (18–22). However, its use as a marker for predicting POP following intracerebral hemorrhage is unprecedented. Studies have confirmed that hypoalbuminemia—reflecting poor nutritional status—contributes to a cascade of underlying mechanisms that elevate POP risk, including impaired organ function, diminished immune competence, reduced phagocytic activity of lymphocytes, decreased antibody-synthesizing enzyme activity, and compromised free radical scavenging capacity (23, 24). Additionally, hypoalbuminemia increases capillary permeability to the interstitial space, rendering patients more susceptible to pleural effusion (25, 26). Pleural effusion elevates pulmonary elastic resistance, impairs diaphragmatic function, and reduces respiratory depth; severe cases may even cause pulmonary atrophy or atelectasis, both recognized risk factors for POP (27, 28). LC and its subsets are key cellular components of the immune system, safeguarding bodily health and preventing disease through cellular immunity and antibody production (29, 30). A low LC may indicate inadequate immune responsiveness, setting the stage for the development of POP. To discuss in general, intracerebral hemorrhage can trigger neurogenic pulmonary edema (NPE) via sympathetic hyperactivity, characterized by heightened pulmonary capillary permeability and alveolar fluid accumulation—directly undermining the lungs’ physical defense barriers (31). A low PNI (defined by hypoalbuminemia and lymphocytopenia) further amplifies this pathological cascade: reduced albumin levels diminish plasma colloid osmotic pressure, exacerbating pulmonary edema (32); concurrently, lymphocytopenia impairs the phagocytic activity of alveolar macrophages, hindering effective clearance of pathogens within the alveoli (33). In the current study, PNI was significantly lower in the POP cohort (38.24 ± 5.27 vs. 44.97 ± 5.01). Through these mechanisms, this reduction may form a “vicious cycle” with NPE, driving up infection risk. Moreover, intracerebral hemorrhage often leads to dysphagia due to cortical or brainstem injury, markedly elevating the risk of aspiration (34). A low PNI exacerbates this risk through two pathways: first, hypoalbuminemia—an indicator of nutritional insufficiency—impairs the reparative capacity of pharyngeal mucosa, leaving aspiration-induced mucosal damage slow to heal and prone to chronic inflammatory foci; second, lymphocytopenia blunts local immune responses, delaying the clearance of oral pathogens introduced via aspiration.

In addition to PNI, advanced age, GCS score, hypoproteinemia, and tracheotomy are also risk factors for POP. Patients with OLD, particularly those with COPD, exhibit irreversible airflow limitation in pulmonary function, leading to significantly impaired clearance capacity of respiratory secretions and subsequent accumulation of sputum in the airways. Meanwhile, aging-related decline in immune function further weakens their anti-infective capacity, rendering them a high-risk group for POP (35). As a core indicator for evaluating the severity of consciousness disturbance, a lower GCS score indicates deeper coma. Such patients are often accompanied by impaired swallowing function and weakened or absent cough reflex, resulting in a significantly increased risk of aspiration of oral secretions and gastric contents, which directly elevates the probability of lower respiratory tract infections (36). Although tracheotomy is a treatment with a lower infection risk than laryngeal tracheal intubation, the disruption of the body’s defensive barrier after tracheotomy, coupled with the loss of the upper respiratory tract’s ability to prevent respiratory pathogens from establishing infection on mucosal surfaces and spreading to the lower respiratory tract, also increases the risk of POP (37, 38).

Previous studies have shown that measures such as adequate perioperative respiratory training, preoperative smoking cessation, preoperative oral hygiene, and early postoperative mobilization can effectively reduce the risk of POP (39–41). However, patients with intracerebral hemorrhage often present with critical conditions and rapid progression. Due to factors such as impaired consciousness and limb dysfunction, they may struggle to tolerate or complete the aforementioned interventions, limiting the availability of clinical preventive measures. The results of this study indicate that PNI not only exhibits good predictive efficacy for postoperative pneumonia in patients with intracerebral hemorrhage but, more importantly, provides a clear direction for clinical intervention—specifically, targeted nutritional support strategies are expected to reduce the risk of postoperative pneumonia. Additionally, PNI calculation relies solely on routine preoperative indicators such as blood routine and liver-kidney function tests, requiring no additional examinations or increased medical costs, which enhances its feasibility and promotional value in clinical practice. The prediction model constructed based on PNI can help clinicians more conveniently identify high-risk populations for postoperative pneumonia, providing references for formulating individualized nutritional support plans and perioperative management strategies. This enables optimization of preventive measures under existing conditions, which holds practical significance for improving the postoperative prognosis of patients with intracerebral hemorrhage. This study has the following limitations: 1. It is constrained by a single-center retrospective design. Data were derived from electronic medical records of a single medical center, influenced by regional diagnostic and treatment protocols, which limits the generalizability of results and may introduce data recording bias. Future research should conduct multicenter prospective cohort studies, incorporating patients from institutions across different regions and with varying levels of medical resources, standardizing data collection criteria to validate the model’s applicability in a broader population. 2. Only static preoperative PNI was used, which fails to reflect dynamic changes in indicators due to postoperative stress, nutritional support, etc., and their real-time impacts. Future studies need to dynamically monitor PNI trends from preoperatively to 2 weeks postoperatively, analyze their association with the timing of POP onset, and explore the role of early postoperative nutritional interventions. 3. The total sample size and the sample size of the POP group are relatively limited, making it difficult to stably validate the role of relevant factors in subpopulations and potentially masking potential interaction effects of some underlying diseases. Future studies should expand the sample size to over 1,000 cases, conduct stratified subgroup analyses, and include more interaction terms between underlying diseases and POP. 4. Although multivariate regression controlled for key variables, unquantified confounding factors (e.g., postoperative nursing quality) remain. Future studies should supplement relevant indicators in the model, clarify mediating effects through path analysis, and explore synergistic effects in combination with microbiome data. In summary, future research should improve the risk prediction system for postoperative pneumonia in intracerebral hemorrhage through multicenter designs, dynamic monitoring, expanded sample sizes, and integration of multi-dimensional indicators, promoting the precision application of PNI in clinical decision-making.

## Data Availability

The original contributions presented in the study are included in the article/Supplementary material, further inquiries can be directed to the corresponding authors.
